# Multivariate Analysis of Influence of Vitamin Intake on Vascular Function Parameters by Sex in the General Spanish Population: EVA Study

**DOI:** 10.3390/nu12030643

**Published:** 2020-02-28

**Authors:** Maria C Patino-Alonso, Marta Gómez Sánchez, Leticia Gómez Sánchez, Rosario Alonso-Domínguez, Natalia Sánchez-Aguadero, Benigna Sánchez Salgado, Emiliano Rodríguez Sánchez, Luis García Ortiz, Manuel A Gómez-Marcos

**Affiliations:** 1 Department of Statistics, University of Salamanca (USAL), IGA Research Group, 37007 Salamanca, Spain; 2 Primary Care Research Unit of Salamanca (APISAL), Biomedical Research Institute of Salamanca (IBSAL), 37005 Salamanca, Spain; martas_111@hotmail.com (M.G.S.); leticiagmzsnchz@gmail.com (L.G.S.); rosa90alonso@hotmail.com (R.A.-D.); natalia.san.ag@gmail.com (N.S.-A.); benissanchez@gmail.com (B.S.S.); emiliano@usal.es (E.R.S.); lgarciao@usal.es (L.G.O.); magomez@usal.es (M.A.G.-M.); 3 Department of Nursing and Physiotherapy, University of Salamanca. 37007 Salamanca, Spain; 4 Health Service of Castilla and Leon (SACyL), 37005 Salamanca, Spain; 5 Iberian network on arterial structure, central hemodynamics and neurocognition, 4800-263 Guimaraes, Portugal, 37005 Salamanca, Spain; 6 Department of Medicine, University of Salamanca, 37007 Salamanca, Spain; 7 Department of Biomedical and Diagnostic Sciences, University of Salamanca, 37007 Salamanca, Spain

**Keywords:** Vitamins Intake, Vascular Function, Canonical Correspondence Analysis, General Population

## Abstract

The influence of vitamin intake on vascular function parameters in the Spanish general population has not been studied. The main objective of this study is to analyze the influence of vitamin intake on vascular function and as a secondary objective the adequacy of vitamin intake in a sample of the Spanish population without previous cardiovascular disease and analyze the differences according to sex. Methods: We included 501 individuals obtained by simple random sampling with replacement (reference population 43,946). The average age was 55.90 ± 14.24 years, 49.70% men. Participants recorded the intake of vitamins using the EVIDENT app, previously validated, during a period of 3 days. Vascular function was assessed by measuring carotid-femoral pulse wave velocity (cfPWV) with the SphygmoCor device, cardio-ankle vascular index (CAVI) with the VaSera device and brachial-ankle pulse wave velocity (baPWV) by using a validated equation. Results: The vitamins with the least adequate intake was vitamin D, less than 5%, and vitamin B_9_, less than 35%. Vitamins with an adequate intake percentage, close to 100%, were B_12_ and B_6_. The multiple regression analysis showed a negative association between cfPWV and vitamin B_2_ in both sexes, and a positive one with retinol in men and B_3_ in women. baPWV was negatively associated with vitamins B_1_ and B_12_ in women and B_9_ in men, while being positively linked with B_6_ in men. CAVI presented a negative association with vitamin D in women. The results were similar in the canonical correspondence analysis. In conclusion, the results of this study suggest that the influence of vitamins on vascular function is not homogeneous and varies according to the parameter analyzed. Thus, in men, vitamins B_2_ and retinol were associated with cfPWV and vitamins B_6_ and B_9_ with baPWV. In women, vitamins B_2_ and B_3_ were related cfPWV, vitamins B_1_ and B_12_ with cfPWV and vitamin D with CAVI.

## 1. Introduction

Arterial stiffness is a manifestation of subclinical organic damage linked to ageing and provides an established marker of cardiovascular disease [1]. Numerous studies have found that the increase in different measures of arterial stiffness, such as the speed of the carotid-femoral pulse wave velocity (cfPWV), brachial-ankle pulse wave velocity (baPWV) and the cardio-ankle vascular index (CAVI) increase morbidity and mortality due to cardiovascular diseases [2,3,4,5]. cfPWV is considered the “gold standard” as a measure of arterial stiffness from the carotid artery to the iliac, reflecting the stiffness in the aorta, and increases with age and blood pressure [6]. Thus, for every meter-per-second increase in cfPWV, the risk adjusted for age, sex and cardiovascular risk factors increased by 14% for total cardiovascular events, and 15% for cardiovascular mortality and all-cause mortality [5].

baPWV is a measure of peripheral arterial stiffness used in Asian countries [7,8] and its clinical utility as a measure of arterial aging was established by Takazawa et al. [9]. CAVI estimates arterial stiffness from the heart to the ankle. It is considered an index of central and peripheral arterial stiffness independent of blood pressure at the time of measurement [10] and can be used as a marker of atherosclerosis and an indicator of lifestyle modification [10,11].

Vitamins are organic compounds necessary for human metabolism. They are involved in proper growth, in the development of the nervous, immune, bone and skin systems. They also play an important role in vision, in the formation of hormones, blood cells, chemicals of the nervous system and genetic material and they intervene in the oxidative stress mechanisms involved in ageing and are thus of crucial importance in healthy living. According to Ames P. [12], in addition to maintaining proper physical fitness, the low hanging fruit in prolonging healthy ageing lies in optimizing vitamin and mineral intake. According to Obeid et al. [13], Vitamin D + Ca + B and D + Ca differentially affect epigenetic age markers, although the effect size appeared to be small after one year. Dietary Reference Intakes (DRIs) are appropriate for assessing the adequacy of nutrient intakes in individuals [14].

Given the above, we know that both arterial stiffness and vitamins are involved in the aging population. Specifically, vitamin deficiency has been associated with cardiovascular disorders and cognitive dysfunction. However, as far as we know, the possible relationship or influence of vitamins on arterial stiffness as a marker of vascular aging has not been studied in the general population. We therefore consider main aim of this analysis to analyze the influence of vitamin intake on vascular function parameters measured with cfPWV, baPWV and CAVI. As a secondary objective, we will evaluate the adequacy of vitamin intake in the general Spanish population free of cardiovascular disease and analyze the differences according to sex.

## 2. Materials and Methods

### 2.1. Study Design

Descriptive transversal study of subjects recruited in the Association between different risk factors and vascular accelerated ageing study (EVA study) (NCT02623894) [15].

### 2.2. Participants

The sample of 43,946 subjects is from an urban population. Random sampling with replacement, stratified by five age groups (ranging from 35.0 to 44.9 years, 45.0 to 54.9 years, 55.0 to 64.9 years, 65 to 74.9 years and over 75 years) and sex was used to select 501 subjects, with approximately 100 in each of the groups, half of each sex. Recruitment was carried out from June 2016 to November 2017. A detailed description of the study methodology, as well as the inclusion and exclusion criteria and the response rate, have been previously published [15]. The recruitment flowchart of the 501 participants is shown in Figure S1 of supplementary material.

### 2.3. Variables and Measuring Instruments: Vascular Function Parameters

#### 2.3.1. Cardio-Ankle Vascular Index (CAVI) and Brachial-Ankle Pulse Wave Velocity (baPWV)

These were measured with a VaSera VS-1500 device (Fukuda Denshi, Tokyo, Japan). The CAVI values were calculated by replacing the stiffness parameter β in the following equation to detect vascular elasticity:

β = 2ρ × 1n/(Ps−Pd) × ln (Ps/Pd) × PWV^2^

where ρ is blood density and Ps and Pd are SBP and DBP in mm Hg, respectively.

baPWV was estimated using the equation baPWV = (0.5934 × height (cm) + 14.4724)/TBA (TBA is the time interval between brachial and ankle waves) [2]. A mean coefficient of variation of the CAVI score of less than 5% is considered small for clinical use and with favorable reproducibility. The measurements were carried out after 10 min of rest and without previous consumption of alcohol or caffeine, following the manufacturer’s instructions [16].

#### 2.3.2. Carotid Femoral Pulse Wave Velocity (cfPWV)

This was measured using the SphygmoCor^®^ device (AtCorMedicalPtyLtd, Head Office, West Ryde, Australia) with the patient supine. cfPWV was calculated by estimating the delay in the pulse wave with respect to the electrocardiogram wave (ECG). The distance measurements were made from the sternal notch to the location where the sensor was placed (carotid and femoral artery) and multiplied by 0.8 [3].

#### 2.3.3. Vitamin Intake

This study analyzed the intake of vitamin A, carotenoids, retinol and vitamin D, considered fat-soluble vitamins and the following water-soluble vitamins C, B_1_, B_2_, B_3_, B_6_, B_9_ and B_12_ and compared them to the DRIs [14]. Participants recorded the intake of vitamins using the EVIDENT application, previously validated [17] during a period of 3 days. The EVIDENT app was developed for smartphones by the computer company CGB and the Primary Care Research Unit of Salamanca (APISAL) (intellectual property registration number 00/2014/2207). The app was configured with the age and sex of each patient.

#### 2.3.4. Physical Activity

Physical activity was assessed with the International Physical Activity Questionnaire—Short Form (IPAQ-SF): The short form (nine items) records the activity of four levels of intensity: (1) intense physical activity, such as aerobics, (2) moderate-intensity activity, such as leisure cycling, (3) walk and (4) sitting for 7 days.

### 2.4. Ethical Principles

All participants provided written informed consent. The study was approved on 4/5/2015 by the Salamanca Ethics Committee for Research with Medicines. The Declaration of Helsinki guidelines were followed throughout the study [18]. The trial was registered in ClinicalTrials.gov with identifier NCT02623894.

### 2.5. Statistical Analysis

Data are expressed as mean and standard deviation (SD), median, ranges and percentages. Normality was assessed using a Kolmogorov–Smirnoff normality test. When comparing categorical variables with each other, the χ2 test was used. Comparisons between groups were performed using Student’s t-test for independent samples or Mann-Whitney-U-Test to evaluate differences by sex within the whole population and within each age group. Analyses of variance (ANOVA) tests with Bonferroni correction for multiple comparisons or Kruskal-Wallis analysis was used to calculate differences among each age group and post-hoc comparisons were made with Dunn’s test. Spearman’s rho correlation coefficient was used to analyze the association between quantitative variables. Multivariate linear regression analysis was used to assess the association of vitamin intake (independent variables) with vascular function parameters (dependent variable) adjusted by age, using the backward stepwise regression method.

To analyze the influence of vitamin intake on vascular function parameters, the restricted ordination method Canonical Correspondence Analysis (CCA) was applied [19]. This method has the advantage of an indirect gradient analysis (ordination) technique, which combines multiple regression analysis with correspondence analysis and thus solves the inconvenience that in the case where the distributions are not homogeneous, we could find ourselves up against the well-known Simpson’s paradox [20], in which the relationship between the variables is changed when the samples are divided into subsamples. Our starting point is two matrices, one containing the information for the subjects related to the items of the three parameters of vascular function (cfPWV, baPWV and CAVI), and the other containing the information related to the intake of the different vitamins (A, D, B_1_, B_2_, B_3_, B_6_, B_9_ and B_12_), carotenoids and retinol. The results are presented by an ordering diagram where the intake scores of vitamins, carotenoids and retinol were plotted by vectors.

For the bilateral contrast of hypotheses, an alpha risk of 0.05 was set as a limit of statistical significance. The data were analyzed using the statistical software SPSS for Windows version 25.0. (IBM, Armonk, New York: IBM Corp). The CANOCO program for Windows version 4.56 was used to perform the CCA.

## 3. Results

### 3.1. Demographic and Clinical Characteristics of the Subjects Included

Analyses included 501 individuals (249 men and 252 women; median age: 55.90 years). Table 1 shows the characteristics of the studied population according to sex. Men showed greater alcohol use than women. Twenty-one percent of women and 17% of men were obese. Vigorous physical activity was performed by 37% of men and 14% of women. The values of vascular function parameters (cfPWV, baPWV and CAVI) were higher in men (*p* < 0.05 in all cases).

### 3.2. Vitamin Intake by Sex and Age

The intake of carotenoids, vitamins B_9_ and B_12_ rose as men’s age increased. The intake of retinol decreased as the women’s age increased. Intake of vitamin A, carotenoids, vitamin C, B_9_ and B_12_ in women is highest at age 51–70. Gender differences were found in the intake of carotenoids, vitamins B_1_, B_3_ and B_12_ (*p* < 0.05), as shown in Table 2.

The vitamins with the least adequate intake were vitamin D and vitamin B_9_ in both sexes. Vitamins with an adequate intake percentage close to 100% were B_3_, B_6_ and B_12_. Significant differences were found in the proportion of participants with a vitamin B_9_ intake over DRIs, in that the highest proportion was found in women over 70 (Table 3).

### 3.3. Association of Vitamins with Vascular Function Parameters

**Figure 1:**
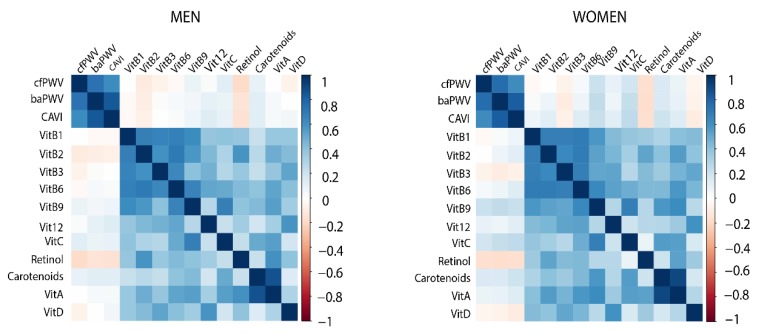
Correlations between the vascular function parameters and vitamins. Positive correlations are displayed in blue and negative correlations in red color. Color intensity is proportional to the correlation coefficients. cfPWV: carotid femoral pulse wave velocity, baPWV: brachial ankle pulse wave velocity, CAVI: cardio–ankle vascular index.

The Spearman correlations between vitamins and vascular function parameters are presented in Figure 1.

Table 4 shows the multiple regression analysis for men and women, considering vascular function parameters as dependent variables and vitamin intake as independent variables. After adjusting for age, cfPWV in men was only associated with retinol (β = 0.001, *p* = 0.023) and vitamin B_2_ (β = –0.620, *p* = 0.031), while baPWV was associated with vitamin B_6_ (β = 0.384, *p* = 0.030) and vitamin B_9_ (β = –0.003, *p* = 0.032). In women, cfPWV maintained an association with vitamin B_2_ (β = –0.475, *p* = 0.009) and vitamin B_3_ (β = 0.023, *p* = 0.027), and baPWV with vitamin B_1_ (β = −0.523, *p* = 0.038) and vitamin B_12_ (β = −0.019, *p* = 0.046), while CAVI is associated with vitamin D (β = −0.046, *p* = 0.007).

### 3.4. Multivariate Characterization of the Relationship between Vitamins and Vascular Function Parameters

In order to study the influence that vitamins have on the parameters of vascular function, CCA was performed. The proportion of explained variance of the first two axes was higher in women (99.7%) than in men (88.9%).

**Figure 2:**
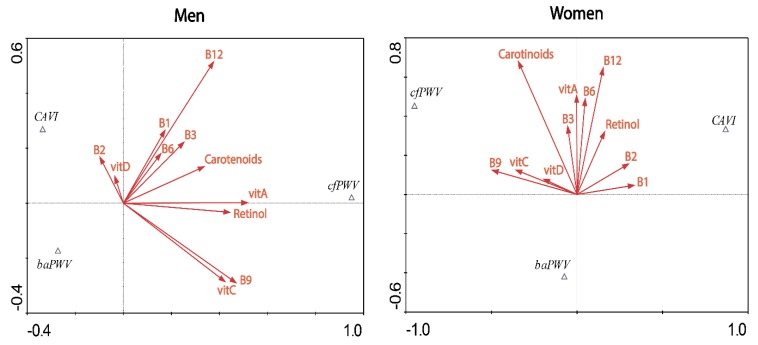
Ordination diagram of the Canonical Correspondence Analysis between vitamins and vascular function parameters. cfPWV: carotid femoral pulse wave velocity, baPWV: brachial ankle pulse wave velocity, CAVI: cardio–ankle vascular index, B1: vitamin B_1_, B2: vitamin B_2_, B3: vitamin B_3_, B6: vitamin B_6_, B9: vitamin B_9_, B12: vitamin B_12_, vitA: vitamin A, vitC: vitamin C, vitD: vitamin D.

The resulting ordination diagram by sex can be seen in Figure 2. The angle between the variables represented by the vitamins allows us to estimate the degree of covariation between them. In men there was a positive association between retinol and vitamin A, between vitamin C and vitamin B_9_ and between vitamins B_1_, B_12_, B_6_ y B_3_. However, a negative relationship was observed between vitamins B_9_ and C with vitamins B_2_ and D. In women we observed a positive association between vitamins C, D and B_9_ and a negative association of these with vitamins B_1_ and B_2_.

To assess the influence of a given vitamin on each of the vascular function parameters, we drew the perpendicular to the vector that joins the vitamin with the origin of coordinates. In men, cfPWV had a positive association with retinol, carotenoids and vitamins A, B_9_ and C, while baPWV was negatively associated with all vitamins. In women, CAVI had a positive association with vitamins B_1_ and B_2_, cfPWV with vitamins D, C, B_9_ and carotenoids, while baPWV was negatively associated with all vitamins.

## 4. Discussion

As far as we know, this is the first study to analyze the relationship between vitamin intake and vascular function, using the most common measures in clinical practice (cfPWV, baPWV and CAVI) to assess it, in a sample of Spanish population free of cardiovascular diseases. In addition to conventional methods for analyzing the relationships between variables, such as correlation and regression analysis, we propose a different methodology based on CCA.

The main findings are that, with the exception of vitamins D and B_9_, vitamin intake is adequate and most participants consume amounts above the DRI limit recommended [14]. Vitamins B_2_, B_3_ and retinol were associated with cfPWV, vitamins B_1_, B_6_, B_9_ and B_12_ with baPWV and vitamin D with CAVI.

In this study, we found a high proportion of subjects to have adequate intake of vitamins A, C, B_1_, B_2_, B_3_, B_6_ and B_12_. These results do not fully agree with the research carried out by Cano–Ibañez et al. [21] in the PREDIMED study, in which it was observed that a considerable proportion of subjects had a deficient intake of vitamins A and E. This could be due to the differences in the age range between the participants of both studies, mainly in women. However, in both studies the proportion of participants with adequate vitamin D intake is low.

Numerous studies show an association of vitamin intake with the prevention of brain ageing, mild cognitive impairment and Alzheimer’s disease, thus reducing all–cause mortality [22,23]. In addition, according to Kang et al. [24], serum vitamin D levels have a beneficial cross–sectional relationship with high density lipoprotein cholesterol levels in both men and women but not with other cardiometabolic risks factors such as blood pressure, blood glucose or other lipid profile determinations, with the ratio between serum 25 (OH) D levels and baPWV mediated by blood pressure. In research involving a sample of 567 patients from the study, Dijk et al. [25] also found no associations of vitamin B levels with PWV. In another study by van Dijk et al. [26], with a two–year intervention in subjects with hyperhomocysteinemia, it was observed that vitamin B_12_ and folic acid did not affect PWV or carotid IMT, nor were any differences in effect found between participants with and without increased arterial stiffness.

In our study, the multivariate CCA analysis showed baPWV to have a negative association in both women and men with the carotenoids, retinol and vitamins considered in the study. These results are in accordance with the work done by Park et al. [27] in a cohort of multicultural communities in Korea, where the intake of β–carotene, vitamin C, folate, or vitamin E was also found to be negatively associated with baPWV.

This research has several limitations. Firstly, the study is cross–sectional in nature, which makes it difficult to establish causal relationships between vitamin intake and vascular structure and function parameters. Secondly, there may be confounding variables that have not been considered in the study. Thirdly, vitamin intake was recorded for a short period of time (3 days) and it cannot therefore be ruled out that such intakes may not be appropriate if considered over the long term.

## 5. Conclusions

The results of this study suggest that the influence of vitamins on vascular function is not homogeneous, and varies according to the parameter analyzed. Thus, in men, vitamins B_2_ and retinol were associated with cfPWV and vitamins B_6_ and B_9_ with baPWV. In women, vitamins B_2_ and B_3_ were related cfPWV, vitamins B_1_ and B_12_ with cfPWV and vitamin D with CAVI.

Prospective studies with a large number of subjects will be necessary to adequately analyze gender differences in the relationship of vitamin intake to vascular function.

## Figures and Tables

**Table 1:** Baseline characteristics of the EVA study participants analyzed by sex.

	Overall (501)	Men (249)	Women (252)	*p*-value
Age, years	55.9 (45.0–66.4)	55.9 (45.2–66.4)	56.1 (44.9–66.0)	0.79
Marital status, *n* (%)				0.016
Single	107 (21.4)	54 (21.7)	53 (21.0)	
Married	331 (66.1)	175 (70.3)	156 (61.9)	
Widowed	31 (6.2)	12 (4.8)	20 (7.9)	
Divorced/separated	32 (6.4)	8 (3.2)	23 (9.2)	
Educational level, *n* (%)				0.49
Primary school	134 (26.7)	66 (26.5)	68 (27.0)	
Secondary school	180 (35.9)	94 (37.8)	86 (34.1)	
Tertiary school	18 (3.6)	6 (2.4)	12 (4.8)	
College university	169 (33.7)	83 (33.3)	86 (34.1)	
Alcohol consumption				<0.001
Teetotaler	250 (49.9)	93 (37.3)	157 (62.3)	
Low risk	201 (40.1)	122 (49.0)	79 (31.3)	
Moderate consumption	37 (7.4)	25 (10.0)	12 (4.8)	
Risk consumption	13 (2.6)	9 (3.6)	4 (1.6)	
Current smoker				0.32
Yes	90 (18.0)	49 (19.7)	41 (16.3)	
No	411 (82.0)	200 (80.3)	211 (83.7)	
Physical activity				<0.001
Low	87 (17.4)	32 (12.9)	55 (21.8)	
Moderate	285 (56.9)	124 (49.8)	161 (63.9)	
Vigorous	129 (25.7)	93 (37.3)	36 (14.3)	
BMI				<0.001
Underweight ≤18.5	7 (1.4)	1 (0.4)	6 (2.4)	
Normal weight = 18.5–24.9	174 (34.9)	71 (28.6)	103 (41.2)	
Overweight = 25–29.9	223 (4.8)	134 (54.1)	89 (35.6)	
Obesity >30	94 (18.8)	42 (16.9)	52 (20.8)	
cfPWV, (m/s)	6.1 (5.1–7.3)	6.3 (5.3–8.1)	5.8 (5.0–7.0)	0.001
baPWV, (m/s)	12.4 (10.9–14.4)	12.7 (11.2–14.6)	12.1 (10.4–14.2)	0.008
CAVI	7.9 (6.9–9)	8.2 (6.9–9.2)	7.7 (6.9–8.8)	0.021
Antidiabetic drugs	35 (7.0)	23 (65.7)	12 (34.3)	0.049
Antihypertensive drugs	96 (19.2)	50 (52.1)	46 (47.9)	0.604
Lipid–lowering drugs	102 (20.4)	49 (48.0)	53 (52.0)	0.707
Type 2 Diabetes	38 (7.6)	26 (68.4)	12 (31.6)	0.016
Hypertension	147 (29.3)	82 (32.9)	65 (25.8)	<0.001
Dyslipidemia	326 (65.1)	162 (49.7)	164 (50.3)	0.905

Values are presented as median (IQR), number and %. Pearson’s chi–square test was performed for categorical variables and Mann–Whitney–U–Test for continuous variables. Abbreviations: IQR: Interquartile Range, EVA: vascular accelerated ageing; *n*: number; BMI: Body Mass Index; cfPWV: carotid-femoral pulse wave velocity; baPWV: brachial-ankle pulse wave velocity.

**Table 2:** Vitamins intake by sex and age.

	Men (*n* = 249)			*p*-value *^a^*	Women (*n* = 252)			*p*-value *^b^*	*p*-value *^c^*
Age Ranges	Years *^1^* ≤ 50 *n* = 99	51 < Years *^2^* ≤ 70 *n* = 100	Years *^3^* > 70 *n* = 50		Years *^1^* ≤ 50 *n* = 100	51 < Years *^2^* ≤ 70 *n* = 102	Years *^3^* > 70 *n* = 50		
**FAT–SOLUBLE VITAMINS**									
Vitamin A (ug/day)	951.91 (673.90–1324.01)	1022.90 (806.41–1308.50)	1104.35 (704.51–1539.73)	0.263	987.21 (750.23–1249.03)	1209.43 (894.12– 1667.48)	1074.08 (788.76–1327.50)	0.003 *	0.086
Carotenoids (ug/day)	2942.00 (1822.68–4476.31)	3387.48 (2464.94–5215.30)	3896.81 (2048.01–5651.49)	0.035 *#	3026.76 (2032.06–4564.49)	4749.78 (2957.60–6611.49)	4048.52 (2366.93–5497.64)	<0.01 *	0.034
Retinol (ug/day)	353.20 (261.18–471.66)	359.17 (225.60–494.01)	312.33 (224.05–385.93)	0.238	364.75 (261.29–502.47)	335.18 (247.60–444.28)	291.69 (210.52–402.65)	0.009 #	0.997
Vitamin D (ug/day)	5.60 (3.51–8.12)	5.94 (4.04–8.42)	5.94 (4.50–7.99)	0.701	5.95 (3.92–8.39)	5.34 (4.07–7.80)	5.13 (3.59–7.73)	0.585	0.635
**WATER–SOLUBLE VITAMINS**									
Vitamin C (mg/day)	1 61.07 ± 92.11	166.48 ±78.07	192.90 ± 89.00	0.104	154.83 ± 69.70	201.09 ± 84.63	198.76 ±70.30	<0.01 *#	0.094
Vitamin B_1_ (mg/day)	1.62 (1.21–2.20)	1.61 (1.28–2.04)	1.71 (1.45–2.07)	0.450	1.47 (1.14–1.87)	1.57 (1.23–1.91)	1.55 (1.32–1.86)	0.429	0.015
Vitamin B_2_ (mg/day)	1.79 (1.42 –2.32)	1.71 (1.36–2.17)	1.83 (1.67–2.21)	0.404	1.64 (1.35–2.09)	1.84 (1.47–2.23)	1.81 (1.56–2.09)	0.107	0.254
Vitamin B_3_ (mg/day)	39.79 ± 13.01	40.14 ± 9.18	41.19 ± 9.45	0.768	38.13 ± 11.54	37.57 ± 11.36	35.14 ± 9.64	0.294	0.004
Vitamin B_6_ (mg/day)	2.46 (1.91–3.20)	2.55 (2.00–3.04)	2.66 (2.14–3.30)	0.504	2.30 (1.87–2.73)	2.50 (2.01–3.11)	2.35 (2.10–2.98)	0.169	0.128
Vitamin B_9_ (ug/day)	305.43 ± 103.36	319.08 ± 100.19	358.59 ± 107.39	0.015#	295.65 ± 108.08	364.23 ± 130.92	354.40 ± 85.32	<0.01 *#	0.185
Vitamin B_12_ (ug/day)	8.45 (5.97–11.84)	8.94 (6.85–13.63)	10.14 (7.35–16.99)	0.050	7.40 (5.61–9.10)	8.83 (6.36–13.09)	7.84 (6.27–10.98)	0.016 *	0.004

Values are presented as median (IQR), mean ± standard deviation; ^a,b^ *p*-value for age; ^c^ *p*-value for sex; 1: participants ≤ 50 Years; 2: participants 51–70 Years; 3: participants > 70 Years. * Difference Years**^1^** and Years**^2^**; # Difference Years**^1^** and Years**^3^**; $ Difference Years**^2^** and Years**^3^**.

**Table 3:** Proportion of participants with a vitamin intake over DRIs by sex and age.

	Men (*n* = 249)				Women (*n* = 252)				
	Years *^1^* ≤ 50 (*n* = 99)	51 < Years *^2^* ≤ 70 (*n* = 100)	Years *^3^* > 70 (*n* = 50)	*p*-value		Years *^1^* ≤ 50 (*n* = 100)	51 < Years *^2^* ≤ 70 (*n* = 102)	Years *^3^* > 70 (*n* = 50)	*p*-value
**FAT–SOLUBLE VITAMINS**					**FAT–SOLUBLE VITAMINS**				
Vitamin A (ug/day) *DRI: 900*	52 (54.5)	60 (60.0)	29 (58.0)	0.489	Vitamin A *DRI:700*	75 (75.0)	85 (83.3)	41 (82.0)	0.271
Vitamin D (ug/day) *DRI: 15,15,20*	2 (2.0)	3 (3.0)	0 (0.0)	0.470	Vitamin D *DRI:15,15,20*	5 (5.0)	5 (4.9)	1 (2.0)	0.239
**WATER–SOLUBLE VITAMINS**					**WATER–SOLUBLE VITAMINS**				
Vitamin C (mg/day) *DRI: 90*	73 (73.7)	83 (83.0)	40 (80.0)	0.160	Vitamin C *DRI:75*	88 (88.0)	95 (93.2)	48 (96.0)	0.109
Vitamin B_1_ (mg/day) *DRI: 1.2*	72 (72.7)	76 (76.0)	44 (88.0)	0.074	Vitamin B_1_ *DRI:1.1*	77 (77.0)	84 (82.3)	41 (82.0)	0.547
Vitamin B_2_ (mg/day) *DRI: 1.3*	82 (82.8)	77 (77.0)	42 (84.0)	0.490	Vitamin B_2_ *DRI:1.1*	88 (88.0)	94 (92.2)	47 (94.0)	0.291
Vitamin B_3_ (mg/day) *DRI: 16*	95 (95.6)	95 (95.0)	48 (96.0)	0.935	Vitamin B_3_ *DRI:14*	99 (99.0)	100 (98.0)	49 (98.0)	0.479
Vitamin B_6_ (mg/day) *DRI: 1.3,1.7,1.7*	94 (94.9)	94 (94.0)	45 (93.8)	0.951	Vitamin B_6_ *DRI:1.3,1.5,1.5*	93 (93.0)	95 (93.2)	44 (88.0)	0.505
Vitamin B_9_ (ug/day) *DRI: 400*	16 (16.2)	22 (22.0)	16 (32.0)	0.084	Vitamin B_9_ *DRI:400*	18 (18.0)	34 (33.3)	35 (70.0)	0.043
Vitamin B_12_ (ug/day) *DRI: 2.4*	95 (96.0)	95 (95.0)	48 (96.0)	0.935	Vitamin B_12_ *DRI:2.4*	98 (8.0)	98 (96.1)	48 (96.0)	0.782

Values presented are percentages of participants with a vitamin intake over DRIs. P values according to age groups. DRI: Dietary reference intake. 1: participants ≤ 50 Years; 2: participants 51–70 Years; 3: participants > 70 Years. DRI: Dietary reference intake. When a single value is displayed, DRI is the same for all groups analyzed. If three values are shown, the first value is for the age group under 50, the second for the group between 51 and 70 and the third for the age group over 70. SOURCES: Dietary Reference Intakes for Vitamin D (1997); Dietary Reference Intakes for Niacin, Vitamin B6, Folate, Vitamin B12 (1998); Dietary Reference Intakes for Vitamin C and Carotenoids (2000); Dietary Reference Intakes for Vitamin A (2001); and Dietary Reference Intakes for Vitamin D (2011). These reports may be accessed via www.nap.edu.

**Table 4:** Association of vitamins with arterial stiffness parameters.

	Men				Women		
	β	(95% CI)	*p*		β	(95% CI)	*p*
**cfPWV**				**cfPWV**			
Age	0.105	0.090; 0.120	<0.001	Age	0.084	0.071; 0.097	<0.001
Retinol (ug/day)	0.001	0.000; 0.001	0.023	Vitamin B_2_ (mg/day)	−0.475	−0.828; −0.121	0.009
Vitamin B_1_ (mg/day)	0.433	−0.061; 0.926	0.085	Vitamin B_3_ (mg/day)	0.023	0.003; 0.043	0.027
Vitamin B_2_ (mg/day)	−0.620	−1.183; −0.058	0.031				
**baPWV**				**baPWV**			
Age	0.120	0.104; 0.136	<0.001	Age	0.159	0.143; 0.174	<0.001
Vitamin B_3_ (mg/day)	−0.023	−0.050; 0.003	0.086	Vitamin B_1_ (mg/day)	−0.523	−1.018; −0.028	0.038
Vitamin B_6_ (mg/day)	0.384	0.038; 0.730	0.030	Vitamin B_3_ (mg/day)	0.024	−0.002; 0.050	0.072
Vitamin B_9_ (ug/day)	−0.003	−0.006; 0.000	0.032	Vitamin B_12_	−0.019	−0.038; 0.000	0.046
**CAVI**				**CAVI**			
Age	0.077	0.067; 0.086	<0.001	Age	0.069	0.061; 0.078	<0.001
Vitamin A (ug/day)	0.001	0.000; 0.001	0.073	Vitamin D (ug/day)	−0.046	−0.080; −0.012	0.007
				Vitamin B_6_ (mg/day)	0.123	−0.016; 0.262	0.083

Multiple regression adjusted by age; cfPWV: carotid femoral pulse wave velocity, baPWV: brachial ankle pulse wave velocity, CAVI: cardio–ankle vascular index, CI: confidence Interval. β: regression coefficient.

## Supplementary Materials

The following are available online at https://www.mdpi.com/2072-6643/12/3/643/s1, Figure S1: Flow diagram of EVA Study.
