# Supplementary figures and images for: Multivariate Analysis of Influence of Vitamin Intake on Vascular Function Parameters by Sex in the General Spanish Population: EVA Study
Source: Nutrients. 2020 Feb 28;12(3):643. doi: 10.3390/nu12030643 (PMC7146244; doi:10.3390/nu12030643)

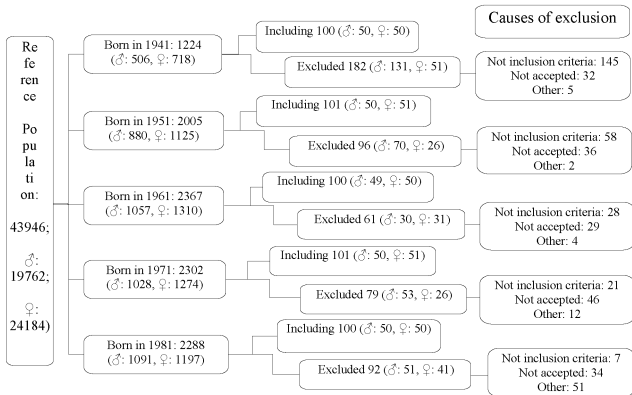

Supplement: Supplementary file 1 [file nutrients-12-00643-s001.pdf]
